# Supplementary material for: Are carbon emissions trading and green financial instruments synergistic? -Comprehensive quantitative research based on content analysis
Source: PLoS One. 2024 Mar 7;19(3):e0298601. doi: 10.1371/journal.pone.0298601 (PMC10919601; doi:10.1371/journal.pone.0298601)
Supplement: S1 Data — (ZIP) [file pone.0298601.s001.zip › Data/地级市Z_F工作报告.zip!/╜¡╬≈/╛┼╜¡╩╨/└┤╫╘╣1⁄2╓┌║┼í╛┬φ┐╦╩2╛▌═°í┐_2018.docx]

各位代表：

　　现在，我代表市人民政府，向大会报告工作，请予审议，并请各位市政协委员和列席会议的同志提出意见。

　　一、2017年工作回顾

　　过去的一年，在省委、省政府和市委的正确领导下，全市上下以迎接党的十九大和学习宣传贯彻党的十九大精神为强大动力，紧紧围绕“九江全面崛起、百姓更加幸福”的目标，攻坚克难，真抓实干，经济社会保持了良好发展态势，较好完成了年初确定的各项目标任务。

　　——经济总量稳步扩张。主要经济指标总量、增幅均位居全省前列。预计全市完成生产总值2400亿元，增长9.3%左右。完成财政总收入461.3亿元，增长11.1%；新增2个县过20亿元、3个县过30亿元。社会消费品零售总额742亿元，增长13%。金融机构本外币各项存款余额2928.8亿元，增长12.2%；各项贷款余额2080亿元，增长18.4%。居民消费价格保持总体稳定。

　　——提质增效成效明显。产业结构进一步优化，三次产业比由7.3：51.9：40.8调整到7.6：50.2：42.2。战略性新兴产业占工业增加值比重提高1.5个百分点。企业效益进一步提升，规模以上工业企业实现利润总额460亿元，增长25%；纳税过亿元工业企业达到7家。

　　——重大项目扎实推进。“重大项目落实年”深入实施，项目建设力度大、推进快、成效好。全市完成固定资产投资2730亿元，增长14%左右。新开工、竣工亿元以上项目282个，完成投资增长70%以上，是近几年来增长最快的一年。

　　——发展动能加速转换。创新创业升温加热，动力活力持续增强。全年科技研发经费投入增长23.8%。完成高新技术产业增加值330亿元，占工业增加值比重提高4.8个百分点。新登记市场主体5.2万户。新增院士工作站2个，总数达到25个。

　　——民生福祉持续增进。民生投入与经济发展保持同步增长，人民群众的获得感进一步增强。全年民生支出占财政总支出的79.3%。城镇、农村居民人均可支配收入分别达到32712元、13312元，分别增长9%、9.5%。

　　过去的一年，我们重点抓了以下七方面工作：

　　（一）突出量质齐升，转型升级步伐进一步加快。“新工业十年行动”深入推进，完成工业固投2130亿元、规模以上工业主营业务收入5600亿元、工业税收200亿元，分别增长14.3%、15%、25%。心连心复合肥一期、天瑞丰收一期、理文包装纸二期、TCL空调、江中食疗产业园、盐津铺子二期、上海永久中部制造基地等一批项目竣工投产；神华电厂一期、赛得利（九江）二期、巨石二期、爱升精密电路板、中船海洋装备和天赐新材料循环产业园等一批项目基本建成。九江石化、九江钢厂、亚东水泥、星火有机硅等一批重点骨干企业效益稳步提升。“两圈两带”旅游格局加快构建，成功承办全省旅游产业发展大会，旅游主要指标位居全省前列。庐山索道建成运营，庐山南门、庐山南山和北山公路等改造完成。庐山西海获评国家体育旅游示范基地创建单位、全国中小学生研学实践教育基地。永修获评全国休闲农业和乡村旅游示范县。“1+7”产业引导基金组建完成。共青私募园区入园基金超过2400家。兴业银行、赣州银行、华融证券开业运营，新增4家企业挂牌“新三板”。全年直接融资突破1000亿元。九江市区域航运中心发展规划编制完成，航运服务中心启动建设。进境木材监管区正式运营，肉类指定口岸建设稳步推进。新增九江-上海始发航线3条，港口货物吞吐量1.2亿吨，集装箱吞吐量33.5万标箱。万达广场、禧徕乐、新天地等一批商业综合体建成运营。颐高、礼涞等电商产业园加快建设，修水获评国家电子商务进农村综合示范县。粮食生产实现“十四连丰”。农村房地一体确权登记发证扎实推进，完成土地流转158.4万亩。新增“两茶一水”种养面积21.5万亩；新增规模以上农业企业24家。庐山云雾茶、宁红茶和瑞昌山药获评全国“最受消费者喜爱农产品区域公用品牌”。彭泽、湖口获评全国渔业健康养殖示范县。

　　（二）突出统筹协调，城乡面貌进一步提升。城镇化率达到53.6%，提高1.4个百分点。九江都市区总体规划和土地利用总体规划调整获得批复。历史文化名城和历史文化街区规划编制完成。中心城区实施重点城建项目138个，完成投资206亿元。赛城湖大桥、兴城大道南段、城西港区三期路网、南组团保障性住房、两湖周边亮化提升等项目建成完工；庾亮南路历史文化街区改造开工建设。深入开展城市管理“打违治乱”专项行动，规范网约车、出租车经营管理，市容市貌和交通秩序进一步好转。武九客专、九景衢铁路建成通车，“动高联运”开通多条高铁线路，九江提前进入“高铁时代”。新改建国省干线公路80公里，都九高速二期加快建设，昌九高速改扩建、良山线公路改造等工程全线开工。“秀美乡村”加快推进，完成新农村整治2419个，“两违”建设得到有效管控。海会镇入选全国第二批特色小镇。新建改造农村公路1200公里、高标准农田26万亩，补充耕地3万亩，完成37座小型水库除险加固和17条中小河流治理。

　　（三）突出改革开放，发展动力进一步增强。蹄疾步稳推进全面深化改革，市本级出台45个具体改革方案，破解了一批长期制约经济社会发展的体制机制障碍，办成了一些多年想办而没有办成的难事。行政区划调整和体制改革取得重大突破，成功撤九江县设柴桑区，创新设立鄱阳湖生态科技城，庐山管理体制改革成效加速显现，八里湖管委会机构成功获批、管理体制进一步理顺，经开区调区扩区基本完成。供给侧结构性改革扎实推进，全面完成“三去一降一补”年度任务，全年降低企业负担近百亿元，房地产市场实现健康平稳发展。启动实施“多证合一、一照一码”登记制度，群众和企业办事更加便捷。深化国有企业改革，“五大集团”完成组建。成功收购联庐公交公司。财税金融、医药卫生、物价、农垦等改革取得新成效。驻浔部队全面停止有偿服务工作稳步推进。成功承办、举办央企入赣投洽会、赣台（九江）经贸文化合作交流会、长江经济带·九江新工业新动能投洽会等活动。引进省外资金845亿元，实际利用外资19.8亿美元，外贸出口292.3亿元。

　　（四）突出创新引领，双创氛围进一步浓厚。制定出台“1+N”科创政策文件，搭建创业孵化平台27个，新增国家级创新载体5个、高新技术企业63家。“树脂基碳纤维研制”获批省级重大科研专项。首个海外（德国）招才引智工作站正式授牌。成功举办第三届全国青年APP大赛和青年企业家发展峰会。湖口科创中心、万其创新创业园建成运营。新增发放“财园信贷通”贷款94.3亿元。积极实施高校毕业生就业创业促进计划，累计发放创业担保贷款17亿元，支持1.4万人创业，带动5.6万人次就业。中船消防公司荣获国家“产学研合作创新与促进奖”。

　　（五）突出绿色发展，生态优势进一步放大。扎实推进生态文明试验区建设，坚决打好环保督察整改攻坚战，一批历史遗留的突出问题得到有效解决。深入推进“河长制”，水生态环境得到改善。全面开展沿江非法码头专项整治，取缔泊位64个，整合泊位76个。花大力气开展城市饮用水水源地保护，河（湖）排污口基本整改到位，河东水厂码头整治、河西水厂引水工程全面完成，提标改造污水处理厂6座，改造污水管网440公里。关停畜禽禁养区养殖场410家，八里湖水面经营权成功收回。城乡环卫一体化体系基本形成，垃圾焚烧发电项目正式启动。园区生态化改造成效明显。九江石化、巨石玻纤获评全国首批“绿色工厂”。河道采砂进一步规范。关停矿山183家。造林绿化17.4万亩，环庐山公路沿线绿化改造提升、市森林博物馆等项目基本完成。新增省级生态乡镇9个、生态村11个。武宁入选国家生态保护与建设典型示范区。瑞昌入选全国农村生活垃圾分类和资源化利用试点。湖口获评国家园林县城。

　　（六）突出执政为民，人民生活进一步改善。夺取了防汛抗洪的全面胜利，涌现出了匡美建、邓旭、程扶摇三位抗洪英雄。脱贫攻坚取得积极进展，预计全年减贫4.5万人、99个贫困村退出，贫困发生率下降1个百分点。省下达民生工程任务全面完成。建成“十个一百”惠民利民项目165个，群众出行、休闲、购物等更加便利。全市改造各类棚户区5.1万户，货币化安置比例达74.4%；改造农村危房8761户。新增城镇就业6.5万人、转移农村劳动力6.1万人。发放教育资助金2亿多元，惠及学生12.4万人；新改扩建学校678所；新招聘中小学教师2299名；共青科教城5所院校全部实现招生。成功举办“庐山论坛-佛教与中国文化峰会”、周敦颐诞辰1000周年纪念活动和首届九派诗会，获评中华诗词之市。新改扩建县级医院8个、村卫计室276所，重大疾病免费救治1.7万人次。全面推行免费婚前医学检查。全民健身活动蓬勃开展，成功举办首届（九江）半程马拉松赛和全国老年人门球交流活动；竞技体育再创佳绩，在第十三届全运会上夺得2枚金牌。城乡低保标准分别提高到530元和305元，惠及24.3万人；实施城乡困难群众医疗救助20.2万人次。困难残疾人生活补贴和重度残疾人护理补贴惠及6.2万人，获得第九届全国残疾人艺术汇演一等奖。全国第三次农业普查工作扎实推进，“七五”普法全面启动。平安九江、法治九江建设不断深入，社会大局保持和谐稳定，公众安全感位居全省前列。德安获评全国平安建设先进县。

　　审计、气象、水文、海关、海事、检验检疫、盐业、通讯、邮政、无线电管理、档案、国安、保密、编制、地方志、外事侨务、民族宗教、驻外联络等工作得到新加强；国防动员、民兵预备役、双拥创建、人民防空、防震减灾、消防等工作取得新成绩；妇女儿童、老龄、志愿者、红十字和慈善等事业实现新发展。

　　（七）突出职能转变，政府自身建设进一步加强。全市上下掀起学习宣传贯彻党的十九大精神热潮，严格落实中央八项规定精神和国务院“约法三章”要求，“两学一做”学习教育向常态化、制度化推进。规范性文件合法性审查和重大行政执法行为报备制度进一步完善,市城区烟花爆竹燃放管理条例施行成效良好。自觉接受人大法律监督、政协民主监督和社会舆论监督，办理人大代表建议和政协提案765件，办结率和满意率均达到100%。持续深化“放管服”改革，市本级行政许可事项、审批窗口授权、行政许可人员到位率均达到90%以上，审批办结时限缩减一半。市政府民声直通车受理群众诉求1万余件，办结率96%。深入开展“不作为、慢作为、假作为、乱作为”等问题专项治理，扎实推进涉农资金监管平台建设，政风行风进一步好转。

　　各位代表，回顾一年来的政府工作，我们深切地感受到：所有成绩的取得，离不开省委、省政府和市委的正确领导，离不开市人大、市政协和社会各界的关心支持，离不开全市人民的共同努力。在此，我代表市人民政府，向各位人大代表、政协委员，向全市人民，向离退休老同志，向各民主党派、工商联、无党派人士、各人民团体，向驻浔人民解放军和武警官兵，向中央和省驻浔单位，向所有关心、支持和参与九江建设的各界朋友，表示衷心的感谢并致以崇高的敬意！

　　各位代表，在总结成绩的同时，我们也清醒地看到，当前我市发展中还存在不少困难和问题，社会主要矛盾已经转化为人民日益增长的美好生活需要和不平衡不充分的发展之间的矛盾。主要表现在：经济总量不大，加快发展的任务仍然艰巨；创新发展能力不强，产业结构不优，转型升级仍然任重道远;经济稳定运行的基础不牢固，实体经济还有不少困难，发展质量和效益仍然有待提高;县域之间、城乡之间发展不平衡，脱贫攻坚任务艰巨，部分群众生活还比较困难，影响社会和谐稳定的因素仍然存在；干部担当创新能力有待进一步增强，行政效能有待进一步提升，发展环境有待进一步优化。对此，我们将高度重视，采取更加有力的措施，努力予以解决。

　　二、2018年工作安排

　　2018年是贯彻党的十九大精神的开局之年，是改革开放40周年，是决胜全面建成小康社会、实施“十三五”规划承上启下的关键一年。党的十九大作出了中国特色社会主义进入新时代的重大论断，确立了习近平新时代中国特色社会主义思想的历史地位，明确了建设社会主义现代化强国的战略安排，我们发展的方向更明；纵观国际国内发展大势，世界经济缓慢复苏，国内经济保持长期向好态势，我们发展的条件更好；长江经济带、长江中游城市群、国家生态文明试验区、鄱阳湖生态经济区、赣江新区、昌九一体化等重大战略汇集叠加，政策红利不断释放，我们发展的机遇更多；尤为重要的是，随着“新工业十年行动”和“重大项目落实年”的深入实施，一批重大项目在加快建设，一批优势产业在加快成长，一批承载平台在加快拓展，我们发展的后劲更足。九江已经站在新的历史起点，必将迈上加速发展的新征程。

　　面向新征程，我们必须牢牢把握新时代，坚定不移加快发展，持续做大总量，大力提升发展质量和效益，着力解决发展不平衡、不充分的问题，努力实现更高质量、更有效率、更加公平、更可持续的发展。必须深入贯彻新理念，将创新、协调、绿色、开放、共享发展理念贯穿发展全过程，推动经济发展质量变革、效率变革、动力变革。必须积极回应新期待，始终坚持以人民为中心的发展思想，坚定不移推进脱贫攻坚，扎实推进民生工程，努力把人民对美好生活的向往变为一件件实事。必须始终牢记新使命，旗帜鲜明讲政治，奋发有为勇担当，驰而不息抓作风，以永不懈怠的精神状态和一往无前的奋斗姿态，为九江谋崛起、为百姓谋幸福。

　　今年政府工作的总体要求是：以习近平新时代中国特色社会主义思想为指导，全面贯彻党的十九大、中央经济工作会议、省委十四届五次全会和市委十一届五次全会精神，按照高质量发展要求，坚持稳中求进工作总基调，坚持新发展理念，坚持以供给侧结构性改革为主线，深入实施新工业十年行动主战略，全面推进“五大组团”建设和“两圈两带”全域旅游发展，扎实开展重大项目服务年活动，着力强新型工业、抓创新开放、建中心城市、促乡村振兴、扬绿色优势、优营商环境、攻精准脱贫，奋力迈出新时代九江全面崛起百姓更加幸福的坚实步伐，为谱写富裕美丽幸福现代化江西的九江篇章扎实开局。

　　全市经济社会发展的主要预期目标是：生产总值增长9%左右；财政总收入增长8%左右；固定资产投资增长12%左右；社会消费品零售总额增长12.5%以上；实际利用外资增长10%左右；规模以上工业增加值增长9%以上；城镇居民和农村居民人均可支配收入分别增长8.5%和9%；居民消费价格总水平涨幅控制在3%以内；完成省政府下达的节能减排任务。

　　为实现上述目标，重点抓好以下十方面工作：

　　（一）坚定不移推进新工业，不断加快全面崛起新步伐。坚持以新工业十年行动主战略为引领，大力发展新经济，促进总量扩大、质量提升。力争规模以上工业主营业务收入、工业固投、工业税收均增长10%以上。

　　加快产业升级。坚持以特色园区为抓手，做大做强特色产业，促进产业集聚、集群、集约发展，加快转型升级步伐。纺织产业园，依托赛得利等龙头企业，加快完善纺纱、织布、印染、成衣产业链，力争主营业务收入达到1100亿元。新材料产业园，做大做强星火有机硅、天赐、晨光等企业，加快完善从研发、中试到生产的产业链，力争主营业务收入达到1100亿元。石化产业园，依托九江石化龙头，加快完善炼化一体化产业链，力争主营业务收入突破900亿元。电子电器产业园，依托巨石玻纤、盛祥电子、生益科技、德宏工业、艾美特、志高、TCL等企业，加快完善粗纱、细纱、电子布、覆铜板、电子电器产业链，力争主营业务收入达到500亿元。新能源产业园，依托北汽、中车、旭阳雷迪、英利等企业，加快完善从引进、配套、生产到使用的新能源产业链，力争主营业务收入达到500亿元。支持装配式建筑、中部LED、卫浴五金、中泰模具等特色产业园加快建设。

　　培育产业龙头。抓好企业培育，推动“企入规、规转股、股上市、育龙头、聚集群”。加快技改扩能，鼓励引导企业通过改工艺、改设备、改产品、改管理等方式，淘汰落后产能，扩大有效供给。全年实施重大技改项目150个以上，推进企业战略性重组30户以上。对表“中国制造2025”，持续推进“两化”融合和智能制造示范点建设。促进品牌提升，鼓励企业抓好质量管理、商标注册、专利申请、产品认证和标准化生产，打响九江产品品牌。力争新增规模以上工业企业150户，新增纳税过亿元工业企业5户以上、过5000万元工业企业8户以上。

　　做优产业平台。强化产业规划引领，优化平台空间布局，每个园区结合自身优势及产业特点，确定1∽2个主导产业进行重点打造。大力推进生态工业园区建设，完善园区公建设施和配套服务，着力解决企业的生产、生活、生根问题，促进产城融合。积极稳妥处置“僵尸”企业，有效盘活闲置厂房和用地等资源，推进园区“二次开发”，新建标准厂房100万平方米以上。推进开发区及工业园区改革创新发展，支持瑞昌创建国家级经开区，支持湖口、彭泽、都昌、永修等园区调区扩区。

　　（二）坚定不移主攻重大项目，不断增强全面崛起新实力。坚持“以项目论英雄”，扎实开展重大项目服务年活动，推动项目建设提速、提质、提效。力争全年实施重大项目753个，完成投资1850亿元。

　　加大项目建设力度。重点推进纤维素纤维、石化PTA和PET、神华电厂、兄弟化工新材料、五星纸业、日昌升、泰盛纸业、一德粮油深加工、金酷科技、北汽、中车等产业项目，加快打造一批新的骨干企业和产业龙头。重点推进安九客专、昌九高速改扩建、昌九发展大道二期等交通项目，完成都九高速都昌至星子段、庐山机场和长江一桥公路桥改造等项目，启动昌九高铁、九岳常、修南铁路等前期工作，增加“动高联运”车次密度。重点推进500千伏九江西、威家220千伏输变电和“永修-武宁-修水”、“蔡岭-都昌”天然气管道等能源项目。

　　创新项目推进机制。在继续推行“五环链接”抓落实、重大项目督查长和“五人小组”项目决策等基础上，实行“分类、分级、分口”调度，切实提高项目推进效率。加大项目督查力度，定期通报项目推进、问题解决和责任落实等情况。探索建立项目建设第三方评估机制，强化督查评估结果运用，严格奖惩问责。

　　优化项目服务环境。把发展环境作为“生命线”来抓，着力构建“亲、清”新型政商关系。做深做细项目前期，积极开展重大项目领办代办服务，着力破解“项目等规划、项目等审批、项目等落地”问题，促进签约项目早落地。全面落实企业降费减负政策，常态化开展“降成本优环境”“政企茶叙会”等活动，持续降低企业运行成本。坚持以诚待商，保持政策的连续性和稳定性，打造守信践诺的诚信环境。深入开展“百企评部门”活动，加大企业投诉的处置督办力度，坚决查处破坏营商环境的人和事，打造公平公正的法治环境，让企业安心生产、放心经营、全心发展。

　　（三）坚定不移推进创新驱动，不断积蓄全面崛起新动能。以创建国家创新型城市和国家知识产权试点城市为契机，深入实施创新驱动发展战略，力争全社会研发投入增长25%以上，新增高新技术企业40家以上，战略性新兴产业占比提高2个百分点以上。

　　做优科创平台。支持企业加强研发平台建设，提高自主创新能力。整合创新资源，在重点企业、产业基地、产业园区加快组建一批重点实验室、企业技术中心、科技企业孵化器等产学研用平台。加快推进鄱阳湖生态科技城、九江产业技术研究院等创新平台建设。支持6354所、九江学院大学科技园、经开区电子电器基地、修水农业科技园争创国家级平台。力争共青城获批国家级高新区。

　　推动协同创新。围绕五大战略性新兴产业，重点在高分子与精细化工、新一代信息技术、人工智能及先进制造、生物医药、绿色食品等领域，实施一批自主创新重大专项和军民融合创新项目。立足传统产业升级，抓好工艺改进、装备更新、产品研发，着力提高产品质量和科技含量。发挥科技创新发展基金撬动作用，培育壮大一批科技型、创新型企业。

　　培育创新人才。强化产业需求导向，深入对接实施省“千人计划”，重点引进一批带资金、带项目、带技术的特殊人才。依托高等院校、科研院所和科技型企业人才平台，加快引进一批有影响、有实力的科技创新团队。实施技能人才培养工程，依托本市职业院校资源，每年培养一批紧缺型和高技能型人才。深入推进“浔才回家”计划，打好乡情牌，吸引一批本土在外英才和成功人士回浔就业创业。大力推进“双百·双千”工程，积极实施人才“尊优计划”，办好创新创业大赛、论坛、培训等活动，进一步浓厚创新创业氛围。

　　（四）坚定不移深化改革开放，不断激发全面崛起新活力。以庆祝改革开放40周年为契机，进一步在深化改革中拓展新空间，在扩大开放中增添新活力。

　　全面深化改革。继续简政放权，深化行政许可“一次办”，积极推进相对集中行政许可权改革，进一步巩固行政审批标准化建设成果，打造“放管服”改革升级版。推进信息网络有效对接，实现数据信息互联共享。深化商事制度、国地税征管体制改革，稳步推进事业单位公车改革和分类改革，加快行政体制、国资国企、财税金融、医药卫生、生态文明、城市管理、文化市场综合执法等领域改革，抓好自然资源和农村房地一体确权登记发证工作，让人民群众在改革中获得实实在在的红利。

　　加强开放合作。深度融入长江经济带和“一带一路”建设，全面提升沿江开放开发水平，打造“江海直达、服务全省、辐射周边”的区域性航运中心，建设万亿临港经济带。加快航运服务中心、彭泽红光综合枢纽港、城西港区上港二期等项目建设，着力构建以水运为核心的集疏运体系。引进培育航运龙头企业，推进“赣沪通、赣浙通”，促进城西港区等转型升级。加快整车、水果等特定进口商品指定口岸申报建设，确保综保区申报成功、肉类指定口岸建设通过国家验收。加大“小散乱”码头整合规范提升力度，强化岸线合理利用与保护。加快瑞昌港区对外开放，力争彭泽港区对外开放早日获批。用好用足国家级赣江新区政策，深入推进昌九一体化，支持沿路县（市、区）改革创新、加快发展。鼓励山区、湖区发挥生态和资源优势，进一步增强开放合作竞争力、吸引力。

　　提升招商水平。实施招商引资升级行动，坚持招商引资与引技引智并重、盘活存量和引进增量并举、专业招商和中介招商并进、重资本招商和轻资产招商并行，不断创新招商方式。聚焦产业抓招商，实施“招大引强行动计划”，深入推进“央企入浔”“外企入浔”“民企入浔”和“浔商回归”工程，继续办好长江经济带·九江新工业新动能投洽会，着力引进一批补链、强链、延链型好项目。创新项目策划包装，制作网上网下“招商地图”。健全招商推进机制，促进项目履约落地。力争全年引进省外资金增长10%。

　　（五）坚定不移打造中心城市，不断拓展全面崛起新空间。坚持以中心城区为引领，以县城集镇为依托，优布局、强功能、聚人气、提品位，进一步增强中心城市的竞争力、带动力和发展力，把九江建设成省域副中心城市和长江经济带重要节点城市。

　　加快建设“五大组团”。全年投入资金200亿元，实施“五大组团”城建项目183个。高效率改造老城区，大力实施新一轮“棚改三年计划”，深入推进边街小巷和沿街立面改造，提升市容市貌，改善人居环境。高质量建设八里湖新区，坚持“八赛联接、九沙联体”，加快打造“城市绿心、城市地标、城市门户”，展现最美新区形象。八里湖片区以中央商务区建设为重点，大力发展金融、科创、文化、旅游、商贸等产业，进一步完善产业体系和服务功能；赛城湖片区重点加快完善规划体系，推进寻阳古城、环湖主干道等项目建设。高目标建设经开区，加大城西港区三期开发力度，加速高端制造和高新技术项目聚集，进一步发挥在全市经济发展中的示范引领带动作用。高标准打造鄱阳湖生态科技城，完成工业综合体项目，加快建设科创中心、芳兰湖湿地公园、地下综合管廊等项目，开工建设物联智慧科技城、科普馆、鄱湖明珠等项目，大力培育和发展高新技术产业，努力打造生态文明样板区和高端产业聚集区。高起点谋划高铁新区，按照“两年拉开框架、五年初步形成、十年基本建成”要求，推进垃圾填埋场迁建工程，加快高铁枢纽工程及周边配套设施建设，再造一个现代化新城区。

　　加快完善交通网络。建成高速公路收费站拆四建二、九湖路、九园路和火车站站房改造等项目，加快推进芳兰大道、琴湖大道、高铁大道、城市快速路等项目，启动八里湖大桥城市之门、沙阎路拓宽等项目前期工作，打通城市内部道路断点堵点，实现新城与老城间、城区与园区间、“五大组团”间快速连接。力争全市升级改造国省道35公里、线路养护大中修105公里。

　　加快塑造城市品牌。突出“山水为体、文化为魂”，大力开展城市“双修”工程，抓好山体、水系、湿地的系统化修复，彰显山水风貌；强化城市设计，加强城市“七线”管控，展现时代新貌。全力推进城市修补，抓好城市绿化、美化、亮化和海绵城市建设，加强历史文化资源保护，不断提升市政配套、公共服务和文化传承功能。大力开展城市“双创”工程，积极创建全国文明城市和国家历史文化名城。加快城市执法体制改革，严格执行市容管理条例，进一步提升市民文明素质。以“两沿两边”为重点，大力开展城乡环境综合整治，确保城乡环境实现大提升。加快县城建设，鼓励各地结合自身资源禀赋和发展基础，因地制宜、突出特色，提升产业和人口承载能力，加快县域经济向城市经济、都市区经济转型，充分发挥美丽宜居宜业县城在乡村振兴中的龙头带动作用。

　　（六）坚定不移推动乡村振兴，不断优化全面崛起新格局。坚持农业农村优先发展，围绕“产业兴旺、生态宜居、乡风文明、治理有效、生活富裕”的总要求，大力实施乡村振兴战略，促进城乡融合发展，夯实全面小康基础。

　　大力发展现代农业。深化农业供给侧结构性改革，扩大农产品有效供给，提高农业综合效益和竞争力。加大耕地保护和补充力度，落实粮食安全责任制，稳定粮食生产能力，新建高标准农田23.5万亩。以“两茶一水”特色产业为重点，全面推进农业产业发展“九大工程”。加快“一县一园”建设，促进资本、科技、人才等要素向园区集聚，力争现代农业示范园区建设取得突破性进展。引进培育农业龙头企业，新增新型经营主体800家、规模以上农业企业30家以上。健全农产品质量安全追溯体系，扩大“三品一标”认证规模，打响一批农业区域公共品牌。

　　着力建设美丽乡村。建立乡村振兴战略规划项目库和资金池，通过撬动金融资本、社会资本，力争五年内全市每年投入100亿元以上，用于乡村振兴、脱贫攻坚和环境整治。按照“点优、线好、面达标”的要求，扎实推进“整洁美丽、和谐宜居”新农村建设，全年建设新农村点4487个，全面完成25户以上贫困村组整治。大力推进“六统·四联创”工作，打造美丽示范县1∽2个、美丽示范乡镇20个、美丽示范村庄200个、美丽示范庭院5万个。加强农民建房管理，保持“两违”管控高压态势。严控农业面源污染，持续推进农村生活垃圾和污水治理。严格落实禁养区、限养区和可养区规划，严防畜禽养殖污染。完善乡村治理体系，建立健全“四有”村庄长效管理机制，培育文明乡风。加大政策支持力度，加快打造云雾、温泉、基金、体育等一批特色小镇。

　　努力加快农民增收。通过调优农业产业结构、推进农业产业化经营、培育发展农业新业态、拓宽农民就业渠道等途径，不断增加农民工资性、经营性和财产性收入，确保小康路上不掉队。深化农村土地制度改革和农村产权制度改革，完善承包地“三权”分置制度，落实第二轮土地承包到期后再延长三十年制度。积极引导土地有序流转，推进农业适度规模经营。扩大政策性农业保险覆盖面，增强农业防御自然灾害能力。支持农民工返乡就业创业，完成“新型职业农民”培育2000人、“一村一名大学生”招录800人、“雨露计划”培训6000人。鼓励发展农村电子商务，建立全市农产品网络交易平台。开展多种形式的“村庄经营”，发展壮大农村集体经济。

　　（七）坚定不移发展现代服务业，不断强化全面崛起新支撑。坚持扬优挖潜，聚焦做大规模、做亮品牌，推动现代服务业总量提升、占比提高。

　　做活旅游经济。以庐山为龙头，加快“两圈”提质扩面、“两带”布点连线，带动全域旅游发展，推动旅游转型升级。推进“庐山天下悠”推介会等签约项目落地；加快环庐山旅游轻轨、庐山石雕公园、庐山演艺中心、环西海旅游公路、庐山西海体育中心、长江风光带、宁州水乡等项目建设；建成归宗景区、庐山·四季花城、牯岭艺术小镇等项目。支持武宁创建国家全域旅游示范区、庐山西海申报5A级景区，启动石钟山创建5A级景区工作。大力实施“旅游+”战略，推动科技、文化、体育与旅游融合发展。加快“智慧旅游”建设，创新旅游宣传营销模式，强化旅游市场综合整治，全面提升旅游服务水平。力争旅游接待总人数和总收入均增长15%以上。

　　发展现代金融。引导金融机构扩大信贷投放，促进金融回归服务实体经济本源。加大金融招商力度，引进金融机构2家。全力推动九江银行、天沅环保、晨光新材料等企业主板上市，加快推进龙开河药业、精达检测等一批企业挂牌“新三板”，支持使用企业债、中期票据、私募债等融资工具，提高直接融资比重。加快引进高水平基金管理团队，推动政府产业引导基金实质性运作。强化“五大集团”专业化投资和资本运营能力，有效盘活政府资产资源，打造全省一流的国资投资运营公司。深入开展“百家银行进千企”等政银企对接活动，创新完善“财园信贷通”“助保贷”等模式,切实缓解中小企业融资难问题。健全保险市场体系，优化保险业务结构，更好发挥保险的经济助推器和社会稳定器作用。加强金融监管，强化信用体系建设，优化金融生态环境，坚决守住不发生区域性、系统性金融风险底线。

　　壮大新兴业态。大力发展现代物流，扎实推进全国物流标准化试点，完善市级公共物流信息平台，加快昌盛物流港等物流园建设，着力构建以港口为核心、水公铁联运为基础的现代物流网络。积极发展电子商务，加快电商服务中心、电商大数据中心、颐高电商产业园和跨境电商产业园等项目建设，做大联盛易佳购、猪八戒网九江园区等平台。加快快递园区建设，推进快递下乡进村，完善快递末端服务。积极发展数字经济，推进“智慧九江”、中邮呼叫中心、腾讯大数据中心等项目建设。大力发展工业设计、商务会展、信息技术、家政服务和健康养老等新兴服务业。

　　（八）坚定不移加快绿色发展，不断厚植全面崛起新优势。扎实推进国家生态文明试验区建设，严守绿色生命线，培育绿色生产力，增强绿色竞争力。

　　巩固提升生态优势。严格落实生态保护红线管制。加强扬尘、油烟、废气、垃圾、秸秆焚烧的监管和整治，加快垃圾无害化处理设施建设，提升空气优良天数比例。加强水行政执法，加快打造“河长制”升级版。按照水体水质“灭五减四增三”的目标，加快推进中心城区水体治理，净化水环境，优化水生态。加强山体保护、地质灾害防治和水土流失综合治理，推进长江崩岸应急治理、昌九高速生态提升和八赛湖控工程等项目。加快生态廊道和绿色矿山建设，造林绿化14万亩，封山育林13万亩。

　　加快发展绿色经济。坚持“减少存量、严控增量”，推动企业减量排放，杜绝“三高一低”项目，构建环境友好的绿色产业体系，争创长江经济带绿色发展示范区。加快发展绿色金融，支持绿色环保、清洁能源和循环经济相关行业发展。推进一批循环低碳试点示范工程，打造一批循环园区。开展能源和水资源消耗、建设用地等总量和强度双控行动，推动资源集约高效利用。

　　严格生态保护制度。全面落实环境保护“党政同责、一岗双责、一票否决”。加强环境执法监管，强化污染源自动监控管理，常态化组织开展环境保护专项行动，持续加大环境违法案件查处力度，让违法者付出沉重代价。严格落实领导干部自然资源资产离任审计制度，完善自然资源有偿使用制度，做好环境保护税征缴工作。推进环保机构垂直管理体制改革。

　　（九）坚定不移以人民为中心，不断满足人民对美好生活的新期待。突出抓重点、补短板、强弱项，加大民生投入力度，精心办好一批社会关注、群众期盼的民生实事。

　　坚决打赢脱贫攻坚战。与全国全省同步建成全面小康社会，是我们的庄严承诺，必须一诺千金。坚持精准扶贫、精准脱贫，注重扶贫与扶志相结合，加快推进“五大工程”建设，确保贫困群众“两不愁、三保障”。把产业扶贫放在首位，支持贫困村发展“一村一品”特色产业，重点推进“农村e邮”精准脱贫和光伏扶贫扩面提升。扎实推进教育精准扶贫，全面落实贫困户子女就学资助政策。加大健康扶贫力度，全面推行贫困人口住院“先诊疗、后付费”，免费办理贫困人口商业补充保险。完成贫困人口易地搬迁5000人。深入开展“三派三服务”活动，提升扶贫帮困实效。全力支持修水、都昌小康攻坚。力争全年5万人脱贫、97个贫困村退出。

　　扎实推进民生工程。加强房地产市场调控，加快建立多主体供给、多渠道保障和“租购并举”的住房制度。改造各类棚户区3万户，改善群众居住条件。大力开展职业技能培训，拓展公益性就业岗位，新增城镇就业6.5万人。稳步推进“公交城市”试点创建工作。加强社会福利和慈善事业发展，健全统筹城乡的社会救助体系。推进宽带提速降费。全面推进农民工工资实名制监管，构建和谐劳动关系。继续实施好“十个一百”惠民利民工程，新建改造绿地小游园、健身小广场、公共停车场、公交候车亭、标准化幼儿园、标准化菜市场、边街小巷、免费公厕、便民放心超市、社区综合服务站等项目200个以上，不断提高人民群众的幸福指数。

　　加快发展社会事业。优先发展教育事业，着力解决中小学生课外负担重、择校热、大班额等突出问题。扩充学前教育资源，加大乡镇公办幼儿园建设力度。完善中心城区教育网点规划布局，加快推进八里湖一小、二小等项目建设，实现全市县域义务教育均衡发展。重视发展特殊教育，积极发展现代职业教育。稳妥做好高考改革工作。支持九江学院教育资源整合，推进共青科教城、永修金蓝领职业培训园建设。深入实施文化惠民工程，加快庾亮南路历史文化街区等项目建设，推动文化事业和文化产业发展，确保通过国家公共文化服务体系示范区验收。广泛开展全民健身活动，积极参加第十五届省运会。加快发展养老事业，推进居家养老服务，探索医养结合发展模式。全面开展优生优育服务活动，提高出生人口素质。实施改善医疗服务行动计划，加快完善分级诊疗体系，合理配置医疗资源，提升基层医疗卫生服务水平。完善城乡居民医疗保险制度，减轻大病患者医疗负担。加强食品药品安全专项整治。推进绿色殡葬改革。启动第四次全国经济普查和第三次全国土地调查工作。

　　全力维护和谐稳定。落实信访责任，有效预防和化解各类社会矛盾。树立安全发展理念，压实安全生产责任，坚决遏制重特大事故发生。持续推进“天网工程”，完善社会治安防控体系建设，严厉打击违法犯罪行为，着力构建平安九江。加快突发事件预警信息发布平台建设。健全应急管理体系，加强救援队伍建设，提高应急处置能力。

　　做好双拥工作，开展“98抗洪”20周年纪念活动，推动军民融合深度发展。充分发挥统计、审计、气象、水文、海关、海事、检验检疫、盐业、通讯、无线电管理、档案、国安、保密、编制、地方志、对台、外事侨务、民族宗教、驻外联络、人民防空、防震减灾、妇女儿童、老龄、残疾人、志愿者、红十字等工作在经济发展和社会建设中的促进作用。

　　（十）坚定不移加强政府自身建设，不断树立实在实干新形象。加快职能转变，推动管理创新，改进工作作风，努力打造“忠诚干净担当、为民务实清廉”的实在实干政府。

　　强化法治思维，推进依法行政。严格在宪法和法律框架内行使职权，做到“法定职责必须为、法无授权不可为”。全面推行法律顾问制度，落实行政执法责任追究制度，规范行政执法行为。自觉接受人大及其常委会的法律监督、工作监督和政协的民主监督，认真办理人大代表建议和政协提案，高度重视人大、政协调研成果的转化运用，广泛听取各界人士的意见建议。深化政务公开，运用全媒体手段及时回应社会关切，不断提高政府工作透明度。

　　强化创新意识，全面增强本领。积极适应新时代发展要求，以增强谋划力、创新力、落实力为重点，加快打造学习型政府。找准上级政策与本地实际的结合点，加强工作研究和对接，创造性地开展工作。找准发展规划与工作计划的结合点，既干好当前，又谋好长远。找准行政手段与市场运作的结合点，激发市场主体活力，更多运用市场化的理念、机制和办法破解发展难题。找准外地经验与九江工作的结合点，积极吸纳消化，大胆借鉴转化。找准试点试验与示范带动的结合点，真正把试点变成亮点，把试验变成示范。

　　强化担当实干，狠抓工作落实。成绩是干出来的，幸福是奋斗出来的。实行“发展项目化、项目责任化、责任具体化”，以项目现场检验实干，以发展成果评价实绩，以群众满意衡量实效。撸起袖子加油干，扑下身子抓落实，坚决防止“以电话调度代替基层调研、以文件落地代替工作落实、以层层批示代替督查指导”。加大懒政怠政问题整治力度，严肃查处一批不担当、不作为、乱作为的干部。健全激励机制和容错纠错机制，为改革创新者撑腰鼓劲，使广大干部的心思集中在“想干事”上、胆识体现在“敢干事”上、能力展现在“会干事”上。

　　强化为民情怀，增进民生福祉。把老百姓的安危冷暖时刻放在心上，以造福人民为最大政绩，围绕群众最关心的教育、就业、收入、社保、医疗、养老、居住、环境等方面的事情，分轻重缓急，多雪中送炭，真正把实事办到人民群众的心坎上。既注重群体的普惠民生，又关心个体的特殊困难；既关心群众的现实利益，又关注群众的切身感受；既考虑为群众做什么，又考虑群众需要我们做什么；既尽力而为，又量力而行，使作出的决策更加符合民主法治，出台的政策更加体现公平公正，发展的过程更加注重安全环保，不断满足人民日益增长的新需求。

　　强化纪律要求，树立良好形象。坚定执行党的政治路线，严格遵守政治纪律和政治规矩。严格落实“一岗双责”，自觉履行主体责任，严守廉洁自律各项规定，清清白白做人、干干净净做事。扎实开展“不忘初心、牢记使命”主题教育，全面抓好中央八项规定实施细则落实，认真执行党政机关办公用房和公务用车管理办法，坚决纠正“四风”不止步。坚持政府过“紧日子”，严控“三公”经费和一般性开支，把每一分钱都用到“刀刃上”，有效防范政府性债务风险。强化工程建设、政府采购、资金使用等重点领域的监管，大力整治损害群众利益的不正之风，以优良政风带动行风民风持续好转。

　　各位代表，深入学习贯彻党的十九大精神，是一项长期的重大政治任务。我们要更加紧密团结在以习近平同志为核心的党中央周围，在省委、省政府和市委的坚强领导下，不忘初心、牢记使命，开拓进取、真抓实干，奋力开启新时代九江全面崛起百姓更加幸福新征程，全力谱写富裕美丽幸福现代化江西的九江篇章！
